# Explainable deep learning framework for fecal contamination detection on chicken eggshells via portable fluorescence imaging under ambient light

**DOI:** 10.1016/j.psj.2026.106868

**Published:** 2026-03-25

**Authors:** Insuck Baek, Lalit Mohan Kandpal, Chansong Hwang, Sookyung Oh, Laura P. Del Collo, Mónica Santín, Bradd J. Haley, Manan Sharma, Jitu Patel, Jianwei Qin, Fartash Vasefi, Moon Kim

**Affiliations:** a Environmental Microbial and Food Safety Laboratory, Agricultural Research Service, United States, Department of Agriculture, Beltsville, MD 20705, USA; b Department of Mechanical Engineering, University of Maryland, Baltimore County, 1000 Hilltop Circle, Baltimore, MD 21250, USA; c SafetySpect, Inc., 4200 James Ray Drive Grand Forks, ND 58202, USA

**Keywords:** Eggshell, Fecal, Explainable AI (XAI), Handheld, Fluorescence imaging, Food safety, Poultry

## Abstract

This study evaluated the efficacy of optimized deep learning architectures using a portable fluorescence imaging device specifically for the in-situ detection of fecal contamination on chicken eggshells to enhance food safety. The research utilized a Contamination and Sanitization Inspection device to establish a comprehensive dataset of fluorescence images, leveraging the spectral characteristics of fecal matter which emits fluorescence in the 600 to 720 nm range. Based on this fluorescence image data set, the study developed high performance models to identify fecal residues across both brown and white eggshells. Experimental results demonstrated that the fluorescence signals of fecal contaminants remain highly stable under ambient lighting, with both the primary mode utilizing 405 nm excitation and the enhance mode utilizing 365 nm excitation achieving Structural Similarity Index Measure (SSIM) values consistently exceeding 0.9200. These metrics confirm that the intrinsic high contrast of fluorescence imaging maintains structural integrity without the need for strict darkroom environments. Through the evaluation of nine distinct neural networks, it was found that the 365 nm excitation effectively suppressed background interference on brown eggs, allowing the lightweight MobileNet architecture to detect fecal contamination with an accuracy of 0.9000. For white eggshells, the 405 nm excitation coupled with the ViT Base 384 model yielded a peak accuracy of 0.9333 in identifying minute fecal traces. The reliability of the detection was further validated through Explainable AI frameworks which confirmed that the classification logic was consistently based on actual contaminated regions marked by fecal residues. These findings provide a robust methodology for leveraging handheld portable fluorescence technology to establish objective standards for detecting fecal contamination in the poultry industry.

## Introduction

Eggs are a globally consumed staple, valued for their rich content of protein, fats, vitamins, and minerals (Atwa et al., 2024). Given this widespread consumption, ensuring the safety and quality of eggs is critical not only for protecting public health but also for maintaining the economic stability of the agricultural sector. However, recent outbreaks highlight persistent vulnerabilities in the supply chain. In June 2025, a major recall involving approximately 1.7 million dozen eggs, nearly 20 million individual units, occurred across at least nine U.S. states due to potential *Salmonella* contamination (Corredor, 2025; U.S. Food and Drug Administration, 2025). This incident resulted in 79 confirmed cases and 21 hospitalizations according to the Centers for Disease Control and Prevention (CDC) (Centers for Disease Control and Prevention, 2025). While no fatalities were reported, the outbreak underscores the severe public health risks posed by *Salmonella* spp. and the substantial economic repercussions for producers and retailers. Compounding these challenges, outbreaks of highly pathogenic avian influenza (HPAI) have caused significant disruptions to poultry production and food supply chains, increased consumer prices (Mitchell and Thompson, 2025). While HPAI is primarily an animal health and supply chain issue rather than a direct foodborne hazard for egg consumers, it nevertheless intensifies pressure on the industry and heightens public awareness of overall food system vulnerabilities.

These incidents emphasize the need to identify and control pathogen transmission vectors within the egg production process. Research identifies fecal residues on the eggshell surface as one of the primary routes for pathogen persistence and transmission (García et al., 2011; Kintz et al., 2024). Surface contamination presents a dual threat by endangering consumer food safety and increasing the risk of cross-contamination among poultry or occupational exposure for workers. To reduce these risks, poultry farms commonly practice regular washing and disinfecting of eggshells to remove contaminants while preserving shell integrity. However, it remains challenging to confirm the effectiveness of these sanitation procedures due to the absence of reliable and standardized methods for inspecting microbial contamination.

Currently, the evaluation of eggshell cleanliness relies predominantly on highly subjective visual and tactile assessments. Visual inspection is primarily employed to detect foreign matter, while tactile examination is used to identify textural irregularities (Gao et al., 2025). While various non-destructive detection techniques have been explored as alternatives, their focus has largely remained on quality grading parameters, such as crack detection, freshness evaluation, or weight prediction, and not specifically on assessing surface cleanliness (Gao et al., 2025; Han et al., 2022; Nasiri et al., 2020; Nematinia and Mehdizadeh, 2018; Sun et al., 2015; Wang et al., 2023; Yang et al., 2023; Zhu et al., 2022). However, surface cleanliness is critical to determining egg safety and consumer protection, playing a central role in preventing pathogen transmission throughout the poultry production chain. Furthermore, many of these modern techniques have only been validated under controlled laboratory conditions, which significantly limits their deployment in real-world agricultural settings. Consequently, there is a critical and unmet need for a practical, accurate, and accessible system that enables inspectors and producers to quantitatively evaluate eggshell cleanliness in situ.

To further illustrate the potential microbial risks associated with unwashed chicken eggs, we conducted a preliminary microbial assay on 15 shell eggs collected from a local poultry farm. Our results revealed that, while *E. coli* and *Salmonella* were not detected, total coliforms were recovered from three eggs and thermotolerant coliforms from two. Notably, one sample exhibited a total coliform count of 3.47 log CFU/egg. These findings quantify the substantial baseline contamination load that sanitation processes must effectively eliminate. Given such high initial contamination levels, any failure or inefficiency in the washing process could pose a significant safety risk. From a strict food safety perspective, there is effectively a zero-tolerance standard for such risks; even a fractional probability of contamination is unacceptable given the potential for widespread public health consequences. Therefore, robust inspection methods are indispensable not only to detect surface residues but to identify and eliminate even the slightest potential hazards, verifying that the microbial burden has been successfully mitigated.

The objectives of this study are as follows: (1) To investigate the impact of ambient illumination on fluorescence imaging quality by acquiring and analyzing images under both controlled darkroom conditions and ambient lighting; (2) To determine the optimal excitation source by comparing the detection accuracy of two distinct LED types; (3) To develop and optimize deep learning models tailored to distinct eggshell pigmentations (white and brown) to ensure detection capabilities; and (4) To validate the reliability of the proposed system by employing Explainable AI (XAI) techniques suited for each architecture. Specifically, by applying Eigen-CAM for CNNs and Attention Map Visualization for ViTs, this study aims to verify that classification decisions are driven by actual fecal contamination rather than background noise, thereby ensuring the interpretability and trustworthiness of the automated inspection.

## Material and methods

### Source of inoculum

Fecal samples were obtained from Ziggy’s Farm, LLC (North Dakota, USA), a facility regulated by the North Dakota Department of Agriculture Meat and Poultry Inspection Program (NDMPIP). In accordance with the Federal Meat Inspection Act (FMIA), the NDMPIP ensures that management practices are consistent with the Guide for the Care and Use of Agricultural Animals in Research and Teaching (Federation of Animal Science Societies, 2020). As the study utilized only excreted materials, no direct animal intervention or handling was performed by the authors. Eight grams of chicken feces were diluted with 72 mL sterile deionized (DI) water in a filtered stomacher bag (GSI Creos Corporation, Japan) and massaged by hand. The filtrate was then passed through a 0.22 µm filter bottle (Corning Incorporated, Oneonta, NY). This filter-sterilized 10% (w/v) stock suspension was further diluted with sterile DI water to 2% and 1% concentrations.

### Egg sample preparation

**Fig. 1:**
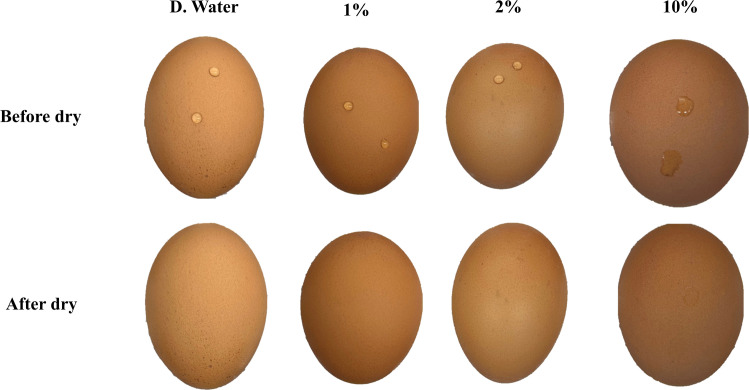
Comparison of two fecal suspension droplets (1%, 2%, and 10%) and DI water (distilled water) on brown eggs before and after drying.

A total of 120 farm chicken eggs (60 brown-shelled and 60 white-shelled) were procured from a local grocery store. To simulate surface contamination, 10 μL of each fecal suspension (1%, 2%, and 10%) was carefully applied to the eggshell surface, with sterile DI water used as a negative control. In the first trial, a single 10 μL droplet was dispensed onto the top surface of each egg. After application, the eggs were dried for 24 hours at 21°C inside a biosafety cabinet. After drying, the applied droplets became scarcely perceptible to the naked eye (Fig. 1). Fluorescence images were then acquired.

Following the first imaging step, each egg was inverted, and two additional droplets (totaling 20 μL) of the same dilution were applied to the opposite surface. After undergoing the same drying process, a second set of images were captured. This procedure enabled the reuse of each egg for dual-sided imaging under controlled conditions. Each egg was individually labeled to track its contamination level. The group sizes were deliberately balanced to ensure consistency across treatment conditions for downstream imaging analysis and model training.

### Handheld fluorescence imaging device

Despite the high sensitivity of fluorescence imaging, its practical application is fundamentally limited by the inherent weakness of fluorescence signals, which are typically orders of magnitude lower in intensity than the excitation source or reflected light (Shashkova and Leake, 2017). To mitigate this, conventional systems strictly require light-tight enclosures or darkrooms to block external photons. However, such physical constraints increase system complexity and hinder seamless integration into dynamic poultry processing lines. Therefore, evaluating detection performance under ambient lighting is essential for developing a robust, field-deployable inspection system.

The Contamination and Sanitization Inspection (CSI) device, collaboratively development by the United States Department of Agriculture Agricultural Research Service and SafetySpect (Active illumination-based multispectral contamination sanitation inspection, system), was employed to acquire fluorescence images. Unlike conventional fluorescence imaging system, the CSI device is capable of capturing fluorescence images under both ambient and dark conditions, differentiating it from general fluorescence imaging systems (technical details are provided elsewhere (Active illumination-based multispectral contamination sanitation inspection, system)).

**Fig. 2:**
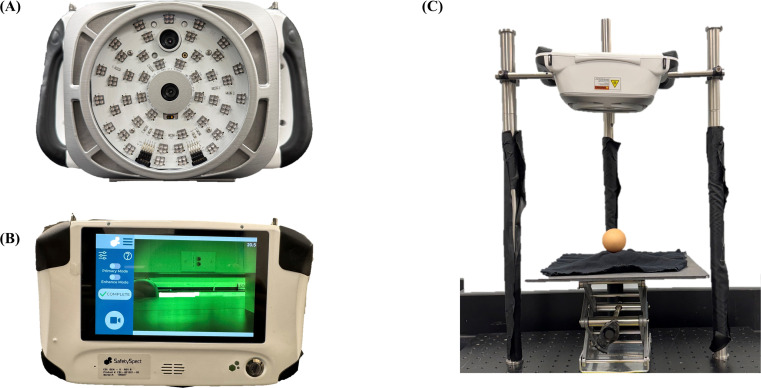
The Contamination and Sanitization Inspection (CSI) device and experimental setup. (A and B) Front and rear view of the CSI device. (C) Stationary experimental setup for fluorescence image acquisition.

As depicted in Fig. 2, which provides a comprehensive visual representation of the device from both its front (Fig. 2A) and rear (Fig. 2B) perspectives, the CSI system comprises two cameras for two types of fluorescence image, two LED light sources (365 nm and 405 nm) for fluorescence excitation, and a single-board computer for device operation. Each camera is specifically paired with one of the two excitation wavelengths. In the primary mode, one camera is excited by 405 nm light to capture two-channel fluorescence images within the 510–560 nm and 665–720 nm spectral bands. Conversely, for the enhance mode, the other camera is excited by 365 nm light to acquire three-channel fluorescence images in the 451–477 nm, 529–555 nm, and 618–660 nm bands.

A design feature is the integration of a specialized optical filter positioned directly above the lens of each camera. This filtering mechanism precisely blocks excitation light, allowing the acquisition of only the pre-defined wavelengths specific to each imaging channel, which is essential for isolating desired fluorescence signals, minimizing background noise, and thereby significantly enhancing image clarity and data reliability. The two distinct types of LEDs are strategically arranged in a uniform circular pattern, as shown in Fig. 2A, to ensure consistent and homogeneous excitation across the imaging area. Furthermore, the device incorporates a display panel on its back, shown in Fig. 2B. This display provides real-time visualization of captured fluorescence images, offering immediate feedback to the user. This real-time imaging capability is invaluable for monitoring experimental progress and ensuring the successful acquisition of high-quality data.

### Acquisition of fluorescence image

Although the CSI device was designed for portability and handheld operation, it was configured as a stationary unit for the purposes of this experiment. This stationary setup was deliberately chosen to eliminate potential movements or fluctuations associated with handheld operation, thereby minimizing variability and maximizing the precision of the acquired fluorescence image data. This approach was crucial for rigorously evaluating the device's fundamental feasibility and assessing its full capabilities, establishing a robust baseline performance before fully leveraging its portable design for future applications. As shown in Fig. 2C, the stationary experimental setup employed for capturing fluorescence images.

For using two different modes, samples were placed at a distance of 225 mm for primary mode and 304 mm for enhance mode as two cameras have different fields of view and prevent fluorescence image saturation. Each sample takes two times in both ambient light and dark since learning processing without ambient light bias.

For each mode, a total of 240 images (60 per class) were collected as detailed in Table 1. The dataset was split into 120 training, 60 validation, and 60 testing images using stratified sampling. To ensure fair and unbiased evaluation, the original images were used in both the validation and test sets without any data augmentation. In contrast, image augmentation was applied to the training set to improve model generalization and robustness.

**Table 1:** Data acquisition configuration for each operation mode under varying lighting and droplet condition.

Mode	Droplet	Light	Brown egg	White egg
Primary	1	On	60	60
		Off	60	60
	2	On	60	60
		Off	60	60
Enhance	1	On	60	60
		Off	60	60
	2	On	60	60
		Off	60	60

Data augmentation is a critical deep learning technique that artificially expands the diversity and size of training datasets by applying various transformations to existing images. This methodology primarily mitigates overfitting by preventing models from merely memorizing training data, instead encouraging the learning of more generalized features. It also effectively alleviates the data scarcity, enabling robust model training even with limited original datasets (Shorten and Khoshgoftaar, 2019). In this study, the Albumentations Python library was employed to perform data augmentation, including HorizontalFlip, VerticalFlip, RandomBrightnessContrast, ShiftScaleRotate, and GaussNoise methods. After augmentation, the total number of training images was 3600.

### Deep learning architectures

**Fig. 3:**
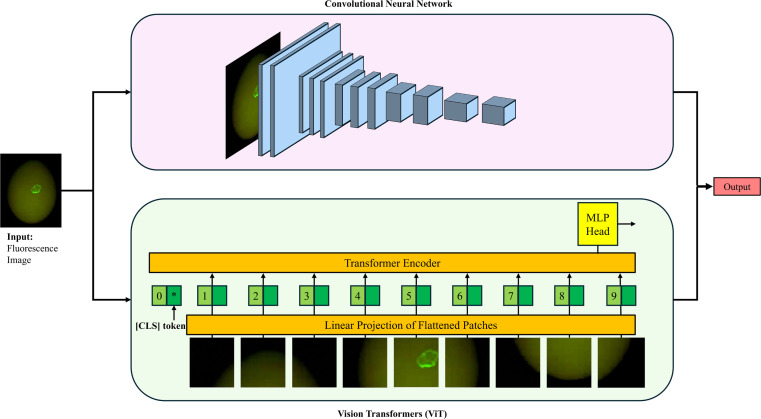
Schematic diagram of the experimental framework. The proposed system evaluates nine distinct deep learning architectures. The models are categorized into CNN-based approaches and Vision Transformer-based approaches.

To comprehensively evaluate the model performance, nine distinct deep learning architectures were implemented and compared, as illustrated in Fig. 3. These models were classified into two primary paradigms: Convolutional Neural Networks (CNNs) and Vision Transformers (ViTs). CNNs utilize convolution operations to extract local spatial features with strong inductive biases, such as translation invariance, which is advantageous for detecting localized surface irregularities. In contrast, ViTs employ self-attention mechanisms to capture global long-range dependencies across the entire image, enabling a comprehensive understanding of the eggshell surface context. By comparing these approaches, this study aims to determine whether the efficiency of local feature extraction or the robustness of global context is more critical for this specific application. Furthermore, this diverse selection allows for a critical evaluation of the balance between inference speed and detection accuracy, providing essential criteria for determining the optimal embedded computing platform specifications required for the handheld device.

#### Convolutional neural networks (CNNs)

Within the CNN category, ResNet-50 and ResNet-101 were selected as baseline models due to their proven stability and deep residual learning capabilities (He et al., 2015). MobileNetV2 was employed to evaluate performance in resource-constrained environments, offering a lightweight architecture specifically optimized for mobile and embedded applications (Howard et al., 2017). Additionally, ConvNeXt was utilized to assess a modernized CNN architecture that incorporates Transformer design principles while retaining the inductive biases of convolutions (Liu et al., 2022).

#### Vision transformers (ViTs)

For the ViT category, the standard Vision Transformer (ViT) series, including ViT-Base (at 224 × 224 and 384 × 384 resolutions) and ViT-Large, was examined to analyze the impact of input size and model scaling on detection accuracy (Dosovitskiy et al., 2020). Furthermore, Data-efficient Image Transformers (DeiT), specifically DeiT-Small, and DeiT-Base, were employed to evaluate the effectiveness of distillation tokens in training high-performance transformers with limited datasets (Touvron et al., 2020).

#### Training strategy and data efficiency

To overcome the challenges associated with the limited size of the fluorescence egg dataset and to ensure robust feature extraction, a transfer learning strategy was adopted for all architectures. Instead of initializing the networks with random weights, we utilized models pre-trained on the ImageNet dataset. This approach leverages vast visual knowledge, enabling the models to recognize fundamental patterns prior to domain-specific training. In addition to the benefits of transfer learning, the intrinsic characteristics of fluorescence imaging further mitigate the need for massive datasets. Unlike standard RGB imaging, which often suffers from low contrast between the eggshell and contaminants, fluorescence imaging generates high-contrast signals where fecal residues exhibit distinct emission intensities against the background. This enhanced signal-to-noise ratio significantly simplifies the feature extraction task for the neural networks. Consequently, the distinct visual patterns reduce the complexity of the underlying data distribution, allowing the models to achieve high convergence and robust generalization even with a relatively smaller number of training samples compared to complex natural scenes.

#### Explainable AI (XAI) framework

To verify the reliability of the classification and ensure that the models base their predictions on actual surface contaminants rather than background artifacts, a comprehensive Explainable AI (XAI) framework was implemented. This framework combines model-agnostic quantitative analysis applied to all architectures with model-specific visualization strategies tailored to the distinct structural mechanisms of CNNs and ViTs. To provide a consistent and quantitative measure of feature importance across all nine models, Local Interpretable Model-agnostic Explanations (LIME) and SHapley Additive exPlanations (SHAP) were universally applied. LIME (Ribeiro et al., 2016) approximates complex deep learning models locally with interpretable linear models by perturbing the input image into super-pixels to identify regions positively contributing to the prediction. Complementing this, SHAP (Lundberg and Lee, 2017), based on cooperative game theory, assigns a Shapley value to each input feature to quantify its marginal contribution to the final classification output, ensuring consistent decision-making logic across both paradigms.

To visualize the spatial focus of the models, interpretability methods specific to each architecture were applied. For CNN-based models, Eigen-CAM (Muhammad and Yeasin, 2020) was employed by computing the principal components of the learned representations in the final convolutional layer. For ViT-based models, Attention Map Visualization (Dosovitskiy et al., 2020) was utilized by extracting self-attention weights from the final transformer block to map the attention intensity allocated to specific image patches (16 × 16).

### Evaluation metrics

To strictly validate the proposed system from image acquisition to final classification, the evaluation framework was divided into three distinct categories: image quality assessment under varying illumination, classification performance of the deep learning models, and computational efficiency for handheld deployment.

#### Image quality assessment

To quantitatively investigate the impact of ambient illumination on fluorescence imaging quality, a comparative analysis was conducted between images acquired under controlled darkroom conditions (reference) and those captured under ambient lighting (test). Two standard metrics were employed to evaluate the degradation in structural integrity and color fidelity caused by external light interference. First, the Structural Similarity Index Measure (SSIM) was utilized to assess the preservation of structural information, including luminance, contrast, and structure. The index ranges from −1 to 1, where the value of 1 indicates perfect structural identity. In this study, a high SSIM value signifies that the fluorescence features of contaminants remain distinguishable from the background even under ambient lighting.

$$\text{SSIM}\left(x,y\right)=\;\frac{\left(2\mu_{x}\mu_{y}+C_{1}\right)\left(2\alpha_{\text{xy}}+C_{2}\right)}{\left(\mu_{x}^{2}+\mu_{y}^{2}+C_{1}\right)\left(\sigma_{x}^{2}+\sigma_{x}^{2}+C_{2}\right)}$$

Where x and y represent the darkroom and ambient images, μ is represents the mean intensity, C represents stabilization constants, and $\sigma^{2}$ represents the variance.

Second, the Color Difference (ΔE)was calculated in the CIELAB color space to evaluate the colorimetric distortion introduced by broadband ambient light. A lower ΔE value indicates that the spectral characteristics of the fluorescence signal are preserved, minimizing the wash-out effect caused by background illumination.

$$(\Delta E=\sqrt{{\left(L_{2}^{*}-L_{1}^{*}\right)}^{2}+{\left(a_{2}^{*}-a_{1}^{*}\right)}^{2}+{\left(b_{2}^{*}-b_{1}^{*}\right)}^{2}}$$

In this equation, $L^{*},\;a^{*},\;\mathrm{and}\;b^{*}$ represent the coordinates in the CIELAB color space, where $L^{*}$ indicates lightness, $a^{*}$denotes the green-red component, and $b^{*}$ represents the blue-yellow component. The subscripts 1 and 2 refer to the values measured under the controlled darkroom condition and the ambient lighting condition, respectively.

#### Classification performance metrics

The classification capability of each deep learning model was evaluated using standard metrics derived from the confusion matrix (accuracy, precision, recall, F1). Given the stringent requirements of food safety, minimizing false negatives is of paramount importance.

Accuracy is defined as the ratio of correctly predicted observations to the total observations. Recall (Sensitivity) is defined as the ratio of correctly predicted positive observations to all observations in the actual class; high recall is critical in this study to ensure that all contaminated eggs are successfully identified. Precision is defined as the ratio of correctly predicted positive observations to the total predicted positives, indicating the reliability of contamination alarms. Finally, the F1-Score is calculated as the harmonic means of Precision and Recall, providing a single score that reflects the overall robustness of the model. The equations for these metrics are as follows:

$$Accuracy=\;\frac{TP+TN}{TP+TN+FP+FN}$$

$$Recall=\;\frac{TP}{TP+FN}$$

$$\text{Precision}=\;\frac{\text{TP}}{\text{TP}+\text{FP}}$$

$$F1-\text{Score}=2\;\times \;\frac{\text{Precision}\;\times \;\text{Recall}}{\text{Precision}+\text{Recall}}$$

In these equations, TP (True Positives) refers to contaminated eggs correctly identified as contaminated, TN (True Negatives) denotes clean eggs correctly classified as clean, FP (False Positives) represents clean eggs misclassified as contaminated (false alarm), and FN (False Negatives) indicates contaminated eggs misclassified as clean (missed detection).

## Results and discussion

### Impact of ambient illumination on fluorescence imaging quality

**Fig. 4:**
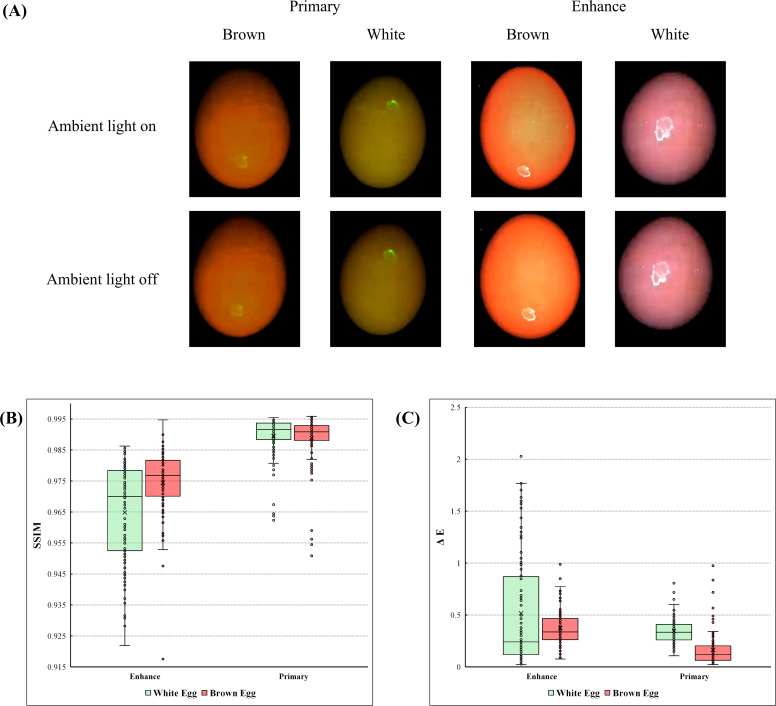
Impact of ambient lighting on the fluorescence imaging performance. (A) Representative fluorescence images of brown and white eggs containing fecal contaminants, captured under 'Primary ' and 'Enhance' excitation modes with ambient light turned on and off. (B) Boxplot comparison of Structural Similarity Index Measure (SSIM) values between ambient light-on and off conditions. (C) ΔE values evaluating color stability against ambient light interference.

Fecal contaminants on the eggshell surfaces are clearly distinguishable in all fluorescence images for both brown and white eggs (Fig. 4A). This effective visualization is attributed to the spectral characteristics of the fecal matter, which emits fluorescence in the 600–720 nm range upon excitation within the 350–430 nm band (Ribeiro et al., 2016). Furthermore, the images exhibit high consistency regardless of the ambient lighting conditions; there are no discernible differences between the images captured with ambient light on versus off (Fig. 4A).

To quantify this visual observation, we analyzed the impact of ambient light on image quality using objective metrics (Fig. 4B). The distribution of SSIM and ΔE values, comparing images captured under ambient lighting against those taken in a controlled darkroom, are illustrated for both white and brown eggs (Fig. 4B and 4C). The evaluation was conducted across two distinct imaging configurations: 'Primary' and 'Enhance', applied to both white and brown eggs.

The primary configuration exhibited exceptional stability, with SSIM values showing a narrow interquartile range and median values approaching 1.0 (Fig. 4B). In contrast, the enhance configuration displayed a relatively broader distribution of SSIM values, indicating a higher sensitivity to external lighting variations. However, despite this increased variance, the absolute SSIM values for the enhance mode remained consistently high, with the majority of data points exceeding 0.92. This suggests that while the enhance mode is slightly more susceptible to ambient noise, the fundamental structural integrity of the image remains sufficiently preserved for accurate feature extraction by deep learning models.

A similar trend was observed in the color difference analysis (Fig. 4C). The primary configuration maintained ΔE values predominantly below 0.5, demonstrating negligible color shift. While the enhance configuration showed a wider dispersion with some outliers reaching higher values, the overall distribution for both configurations remained well within the range of low color distortion. This indicates that the spectral characteristics of the fluorescence signal are effectively preserved without significant signal degradation, even when the enhance mode's higher sensitivity captures background ambient light.

### Performance metrics of detection models

The classification performance of the deep learning models was evaluated under the primary (405 nm) and enhance (365 nm) modes. While key findings are summarized in Table 2, Table 3, comprehensive metrics, including precision and recall, are detailed in Supplementary Tables S1–S4. These results reveal that the interaction between the excitation light and eggshell pigmentation plays a decisive role in determining the optimal model architecture.

**Table 2:** Classification performance of deep learning models under the primary imaging configuration. The evaluation metrics (F1-score, Accuracy) are presented for validation and test datasets, stratified by Brown and White eggshells.

	Brown Egg				White Egg			
Model	Validation		Test		Validation		Test	
	F1	Acc.	F1	Acc.	F1	Acc.	F1	Acc.
ConvNeXt	0.8354	0.8333	0.7729	0.7833	0.9499	0.9500	0.8625	0.8667
MobileNet	0.8177	0.8167	0.7658	0.7667	0.8831	0.8833	0.8049	0.8000
ResNet-50	0.8559	0.8667	0.7133	0.7333	0.9348	0.9333	0.9165	0.9167
ResNet-101	0.8175	0.8167	0.8146	0.8167	0.9019	0.9000	0.8477	0.8500
DeiT-B.	0.8824	0.8833	0.7955	0.8000	0.8993	0.9000	0.8854	0.8833
DeiT-S.	0.8321	0.8333	0.7935	0.8000	0.9010	0.9000	0.8686	0.8667
ViT-B.−224	0.8529	0.8500	0.8346	0.8333	0.9001	0.9000	0.8999	0.9000
ViT-B.−384	0.9160	0.9167	0.7985	0.8000	0.9002	0.9000	0.9321	0.9333
ViT-Large	0.8665	0.8667	0.8992	0.9000	0.8995	0.9000	0.8344	0.8333

Acc. = Accuracy.

F1 = F1 Score.

B.=Base.

S.=Small.

ViT = Vision Transformer.

**Table 3:** Classification performance of deep learning models under the enhance imaging configuration. The evaluation metrics (F1-score, Accuracy) are presented for validation and test datasets, stratified by Brown and White eggshells.

	Brown Egg				White Egg			
Model	Validation		Test		Validation		Test	
	F1	Acc.	F1	Acc.	F1	Acc.	F1	Acc.
ConvNeXt	1.000	1.000	0.8694	0.8667	0.8839	0.8833	0.8661	0.8667
MobileNet	1.000	1.000	0.8985	0.9000	0.7779	0.7833	0.7914	0.8000
ResNet-50	1.000	1.000	0.7957	0.8000	0.7627	0.7667	0.7300	0.7500
ResNet-101	1.000	1.000	0.8665	0.8667	0.7524	0.7500	0.8144	0.8167
DeiT-B.	1.000	1.000	0.8138	0.8167	0.9332	0.9333	0.9166	0.9167
DeiT-S.	1.000	1.000	0.7816	0.7833	0.9163	0.9167	0.8331	0.8333
ViT-B.−224	1.000	1.000	0.8352	0.8333	0.9493	0.9500	0.8825	0.8833
ViT-B.−384	1.000	1.000	0.8633	0.8667	0.9139	0.9167	0.9166	0.9167
ViT-Large	1.000	1.000	0.7503	0.7500	0.9665	0.9667	0.8839	0.8833

Acc. = Accuracy.

F1 = F1 Score.

B.=Base.

S.=Small.

ViT = Vision Transformer.

Under 405 nm excitation (Primary mode), classification performance was governed by both spectral contrast and the model's capacity to resolve fine spatial details. White eggshells exhibit strong intrinsic autofluorescence peaking at approximately 454 nm (blue region) (Ostertag et al., 2019). Although this signal is spectrally distinct from the red fluorescence (600–720 nm) emitted by fecal contaminants (Kim et al., 2003), it can introduce background noise. In primary mode, signal acquisition was limited to two specific regions, effectively eliminating interference from the 454 nm autofluorescence of the white eggshell. However, spectral separation alone does not fully explain the observed peak performance; preservation of spatial information proved equally critical. Notably, ViT-Base-384 achieved the highest classification accuracy (0.9333), outperforming the standard resolution ViT-Base-224, which achieved an accuracy of 0.9000. This performance difference highlights the importance of high input resolution for preserving fine-grained spatial features associated with minute contaminants that may otherwise be lost in lower-resolution feature maps.

This reliance on spatial detail is further supported by the performance of the CNN architectures. ResNet-50 achieved a high accuracy of 0.9167, noticeably outperforming the deeper ResNet-101, which scored 0.8500. This counter-intuitive result suggests that for high-contrast targets such as contaminants on white shells, excessive network depth can be detrimental. Although deeper networks like ResNet-101 possess larger receptive fields, their aggressive feature abstraction tends to erode fine-grained local signals of minute contaminants. In contrast, ResNet-50 strikes an optimal balance, leveraging the inductive bias of CNNs to extract sharp local spatial features without over-smoothing the small anomalies.

In contrast, for brown eggs, the 405 nm excitation resulted in a significantly lower signal-to-noise ratio (SNR). This is because 405 nm targets the Soret band of Protoporphyrin IX (PPIX), causing the pigments distributed across the entire shell to fluoresce intensely in the red region (Ostertag et al., 2019). Since fecal contaminants also emit fluorescence in this same red spectral band, the background shell fluorescence acts as direct optical interference. Crucially, this challenging optical environment highlighted the necessity of high model capacity. The impact of spectral overlap is clearly reflected in the performance disparity within model families. Among CNNs, the deeper ResNet-101 achieved an accuracy of 0.8167, significantly outperforming the shallower ResNet-50. This suggests that under high-interference conditions, increased network depth is required to learn the complex non-linear boundaries needed to disentangle the contaminant signal from the background noise. This trend culminates in the ViT-Large model, which possesses the highest computational burden; it achieved the peak accuracy of 0.9000. These results collectively indicate that when spectral signals overlap, the computational capacity (depth and parameter count) of the model becomes the determining factor for accurate detection.

Switching the excitation source to 365 nm fundamentally altered the feature space, leading to a remarkable shift in model performance. The most significant finding was observed in brown eggs, where the lightweight MobileNet achieved a surge in the accuracy to 0.9000, drastically outperforming its 405 nm result of 0.7667.

This performance leap is attributed to the optical properties of the brown eggshell. Reflectance studies indicate that brown eggshells have significantly higher reflectance at 365 nm compared to the strong absorption peak at the 410 nm Soret band (Ostertag et al., 2019). This higher reflectance implies reduced photon absorption by the shell, thereby minimizing background fluorescence. Consequently, the detection mechanism at 365 nm shifts to rely on fluorophore density. While the shell possesses only a thin pigment layer, fecal contaminants consist of concentrated fluorophore aggregates (Gorji et al., 2022). Under 365 nm excitation, where the background is optically suppressed, the high concentration of fluorophores in the feces still emits sufficient photon flux. This creates a high Signal-to-Background Ratio and effectively causes contaminants to pop out against the egg background. This optical optimization simplifies feature extraction and allows even resource-constrained CNNs to identify contaminants with high precision.

However, an interesting reversal was observed for white eggs under 365 nm, where the deeper ResNet-101 (0.8167) outperformed ResNet-50 (0.7500). This shift indicates a significant increase in task complexity. Analogous to the spectral interference observed in brown eggs under 405 nm, the 365 nm excitation on white eggs creates a high-noise environment. The imaging system in enhance mode captures the intrinsic autofluorescence of the white eggshell at 454 nm, which becomes intensely activated under UV light. This strong background signal overlaps with the contaminant features, causing them to become subtle and indistinct. In this challenging scenario, the shallower ResNet-50 underfitted due to the spectral confusion, while the deeper architecture of ResNet-101 provided the necessary computational capacity to disentangle these fainter, abstract feature representations from the intense background fluorescence.

Collectively, these findings indicate that the optimal inspection strategy must be adaptive and consider both the eggshell variety and the available hardware resources. For brown eggs, enhance mode is unequivocally superior. By leveraging the density-based optical contrast that suppresses background noise, it enables the lightweight MobileNet to achieve a high accuracy of 0.9000. This makes it the ideal choice for resource-constrained handheld devices.

Conversely, for white eggs, primary mode is essential to secure sufficient spectral contrast. In this configuration, model selection requires balancing maximum precision against computational efficiency (see Supplementary Table S5 for input resolution, parameters, and floating-point operations (FLOPs) of all models). While ViT-Base-384 demonstrated the highest sensitivity with an accuracy of 0.9333, ResNet-50 offered a highly competitive performance of 0.9167. For real-time applications on edge processors, ResNet-50 presents a more pragmatic solution because it provides robust detection with significantly lower latency and memory usage compared to ViT. Therefore, future development of portable inspection systems should prioritize matching the model architecture to the hardware specifications by deploying efficient CNNs like MobileNet and ResNet-50 for handheld units while reserving high-capacity Transformers for high-performance computing environments.

### Explainability analysis of deep learning models

**Fig. 5:**
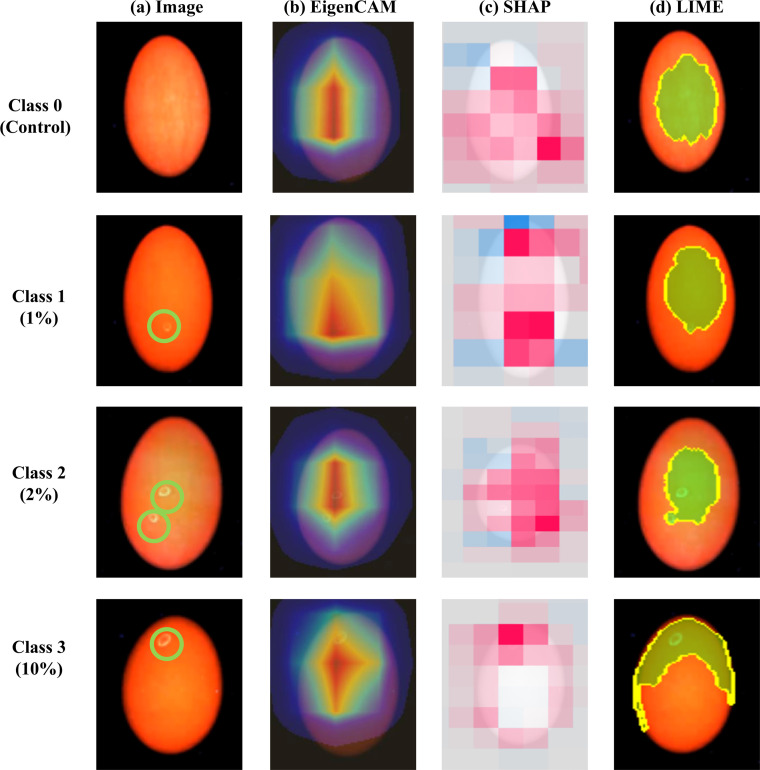
Explainable AI (XAI) visualization of the MobileNet model for brown eggshells using the enhance mode. The rows correspond to contamination severity levels ranging from Class 0 to Class 3. The columns display: (a) original fluorescence images with ground truth contamination marked with green circles; (b) Eigen Class Activation Mapping (EigenCAM) maps highlighting the model's focus areas; (c) SHapley Additive exPlanations (SHAP) summary plots where red pixels indicate positive contributions to the classification; and (d) Local Interpretable Model-agnostic Explanations (LIME) outlining the most significant super-pixel with yellow boundaries.

**Fig. 6:**
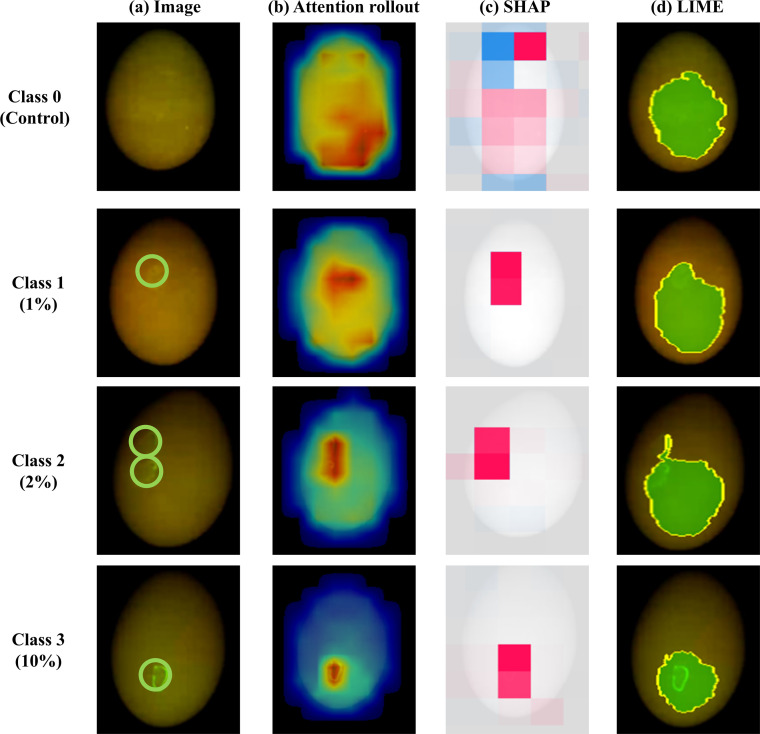
Explainable AI (XAI) visualization of the ViT-base-384 model for white eggshells using primary mode. The rows correspond to contamination severity levels ranging from Class 0 to Class 3. The columns display: (a) original fluorescence images with ground truth contamination marked with green circles; (b) attention rollout maps highlighting the model's focus areas; (c) SHapley Additive exPlanations (SHAP) summary plots where red and blue pixels indicate positive and negative contributions to the classification, respectively; and (d) Local Interpretable Model-agnostic Explanations (LIME) outlining the most significant super-pixel with yellow boundaries.

To ensure a focused and meaningful interpretability analysis, the scope of the evaluation was narrowed from the nine initially tested models to the single best-performing architecture for each eggshell variant. Specifically, the XAI implementation was conducted exclusively on the enhance mode utilizing MobileNet for brown eggshells and the primary mode based on ViT-base-384 for white eggshells (Fig. 5, Fig. 6). These selected models were then subjected to distinct XAI techniques tailored to their respective architectures to visualize the specific features contributing to their high-performance classification. For the CNN-based MobileNet applied to brown eggshells, EigenCAM was utilized to project class activation maps, while for the ViT-base-384 applied to white eggshells, Attention Rollout maps were extracted to visualize the self-attention mechanisms inherent in the transformer blocks. In addition to these architecture-specific methods, SHAP and LIME were applied to both models to provide a comparative and model-agnostic perspective.

The comparative analysis across different contamination levels from Class 0 to Class 3 reveals that both the MobileNet and ViT-base-384 models consistently prioritize the actual contaminated regions, marked by the green circles in the original fluorescence images, as the primary diagnostic evidence. For brown eggshells, the EigenCAM heatmaps display concentrated high activation regions that precisely overlap with the localized contamination. Similarly, for white eggshells, the Attention Rollout maps pinpoint the exact location of defects through high-weight attention zones, demonstrating that the transformer-based model effectively captures the spatial dependencies of anomalous features.

The SHAP analysis further aligns with this spatial consistency, employing 2,000 iterations per explanation to ensure statistical convergence. The resulting SHAP maps show that positive SHAP values, indicated by red pixels, are predominantly clustered within the contaminated zones of both eggshell types. This alignment confirms that these specific pixel groups are the most influential factors in increasing the model's confidence for the respective contamination class. Furthermore, the LIME results offer a granular validation by highlighting the single most significant super-pixel region that the model deems critical for its prediction. In this study, the LIME analysis was also conducted with 2,000 samples to stabilize the local surrogate model, and the visualization was restricted to the top-performing region to clearly isolate the most dominant feature. The yellow boundaries generated by LIME consistently encapsulate the contaminated areas across all classes, mirroring the visual logic used by human experts for identification.

The convergence of these independent methodologies, ranging from gradient-based and attention-based visualizations to additive feature attribution, suggests that the optimized models have developed a robust and interpretable understanding of the data. Notably, the high degree of overlap between the XAI-detected regions and the ground-truth contamination across both brown and white eggshells validates the scientific reliability of the proposed inspection system. These findings confirm that the superior performance of the selected architectures is driven by feature learning rather than background artifacts, providing valuable insights for their deployment in safety-critical inspection tasks.

## Conclusion

This research presents a practical and scalable framework for the quantitative assessment of fecal contamination on eggshells, bridging the gap between laboratory-grade analysis and real-world application. By integrating portable fluorescence imaging with optimized deep learning architectures, we demonstrated that the proposed system achieves robust detection accuracy even under standard ambient lighting, eliminating the need for strict darkroom conditions typically required by conventional fluorescence inspection protocols. This environmental adaptability is a critical factor that ensures the system's viability for monitoring across diverse agricultural settings.

A key contribution of this work is the validation that tailoring deep learning strategies to specific optical configurations, specifically by matching excitation wavelengths and model architectures to eggshell pigmentation, is essential for maximizing detection precision. Furthermore, the efficiency analysis confirms that these high-performance models function within the computational constraints of embedded systems, verifying the feasibility of a handheld device for real-time operation. The incorporation of Explainable AI (XAI) further substantiates the system’s reliability by providing transparent visual evidence that aligns with human expert logic, thereby fostering trust in automated decision-making processes.

Ultimately, this technology offers a transformative solution for the poultry supply chain by replacing subjective visual and tactile inspections with an objective, standardized screening method. By enabling the early and accurate identification of pathogen vectors, this system holds significant potential to enhance overall food safety protocols, mitigate food safety risks, and support the economic stability of the egg production industry.

## CRediT authorship contribution statement

**Insuck Baek:** Writing – review & editing, Writing – original draft, Methodology, Investigation, Formal analysis, Data curation, Conceptualization. **Lalit Mohan Kandpal:** Writing – review & editing, Methodology, Investigation, Formal analysis, Data curation. **Chansong Hwang:** Writing – review & editing, Validation, Investigation, Formal analysis, Data curation. **Sookyung Oh:** Writing – review & editing, Methodology, Formal analysis, Data curation. **Laura P. Del Collo:** Writing – review & editing, Methodology, Formal analysis, Data curation. **Mónica Santín:** Writing – review & editing, Supervision, Conceptualization. **Bradd J. Haley:** Writing – review & editing, Supervision, Methodology, Data curation. **Manan Sharma:** Writing – review & editing, Supervision, Methodology, Data curation. **Jitu Patel:** Writing – review & editing, Supervision, Methodology, Data curation. **Jianwei Qin:** Writing – review & editing, Visualization, Validation. **Fartash Vasefi:** Writing – review & editing, Resources, Conceptualization. **Moon Kim:** Writing – review & editing, Writing – original draft, Supervision, Project administration, Funding acquisition, Conceptualization.

## Disclosures

The authors declare the following financial interests/personal relationships which may be considered as potential competing interests:

Insuck Baek has patent issued to Safetyspect Inc and US Department of Agriculture. Moon S. Kim has patent issued to Safetyspect Inc and US Department of Agriculture. Fartash Vasefi has patent issued to Safetyspect Inc and US Department of Agriculture. Jianwei Qin has patent pending to Safetyspect Inc and US Department of Agriculture. If there are other authors, they declare that they have no known competing financial interests or personal relationships that could have appeared to influence the work reported in this paper.

## Appendix Supplementary materials

Image, application 1

## Footnotes

Supplementary material associated with this article can be found, in the online version, at doi:10.1016/j.psj.2026.106868.
