# Supplementary material for: Explainable deep learning framework for fecal contamination detection on chicken eggshells via portable fluorescence imaging under ambient light
Source: Poult Sci. 2026 Mar 25;105(7):106868. doi: 10.1016/j.psj.2026.106868 (PMC13091751; doi:10.1016/j.psj.2026.106868)
Supplement: Supplementary file 1 [file mmc1.docx]

Supplementary materials

Supplementary Table S1. Detailed classification performance metrics for brown eggs under Prime mode (405 nm excitation), including precision, recall, and F1-score.

|  | Validation | | | | Test | | | |
| --- | --- | --- | --- | --- | --- | --- | --- | --- |
|  | Precision | Recall | F1 score | Accuracy | Precision | Recall | F1 score | Accuracy |
| ConvNeXt | 0.8406 | 0.8333 | 0.8354 | 0.8333 | 0.8003 | 0.7833 | 0.7729 | 0.7833 |
| MobileNet | 0.8414 | 0.8167 | 0.8177 | 0.8167 | 0.7708 | 0.7667 | 0.7658 | 0.7667 |
| ResNet-50 | 0.8906 | 0.8667 | 0.8559 | 0.8667 | 0.7491 | 0.7333 | 0.7133 | 0.7333 |
| ResNet-101 | 0.8523 | 0.8167 | 0.8175 | 0.8167 | 0.8314 | 0.8167 | 0.8146 | 0.8167 |
| DeiT-Base | 0.9081 | 0.8833 | 0.8824 | 0.8833 | 0.7955 | 0.8000 | 0.7955 | 0.8000 |
| DeiT-Small | 0.8353 | 0.8333 | 0.8321 | 0.8333 | 0.8149 | 0.8000 | 0.7935 | 0.8000 |
| ViT-Base-224 | 0.8653 | 0.8500 | 0.8529 | 0.8500 | 0.8407 | 0.8333 | 0.8346 | 0.8333 |
| ViT-Base-384 | 0.9174 | 0.9167 | 0.9160 | 0.9167 | 0.7996 | 0.8000 | 0.7985 | 0.8000 |
| ViT-Large | 0.8683 | 0.8667 | 0.8665 | 0.8667 | 0.9069 | 0.9000 | 0.8992 | 0.9000 |

Supplementary Table S2. Detailed classification performance metrics for brown eggs under Prime mode (405 nm excitation), including precision, recall, and F1-score.

|  | Validation | | | | Test | | | |
| --- | --- | --- | --- | --- | --- | --- | --- | --- |
|  | Precision | Recall | F1 score | Accuracy | Precision | Recall | F1 score | Accuracy |
| ConvNeXt | 0.9527 | 0.9500 | 0.9499 | 0.9500 | 0.8653 | 0.8667 | 0.8625 | 0.8667 |
| MobileNet | 0.8856 | 0.8833 | 0.8831 | 0.8833 | 0.8250 | 0.8000 | 0.8049 | 0.8000 |
| ResNet-50 | 0.9392 | 0.9333 | 0.9348 | 0.9333 | 0.9215 | 0.9167 | 0.9165 | 0.9167 |
| ResNet-101 | 0.9090 | 0.9000 | 0.9019 | 0.9000 | 0.8578 | 0.8500 | 0.8477 | 0.8500 |
| DeiT-Base | 0.9015 | 0.9000 | 0.8993 | 0.9000 | 0.8906 | 0.8833 | 0.8854 | 0.8833 |
| DeiT-Small | 0.9052 | 0.9000 | 0.9010 | 0.9000 | 0.8745 | 0.8667 | 0.8686 | 0.8667 |
| ViT-Base-224 | 0.9080 | 0.9000 | 0.9001 | 0.9000 | 0.9018 | 0.9000 | 0.8999 | 0.9000 |
| ViT-Base-384 | 0.9071 | 0.9000 | 0.9002 | 0.9000 | 0.9330 | 0.9333 | 0.9321 | 0.9333 |
| ViT-Large | 0.9070 | 0.9000 | 0.8995 | 0.9000 | 0.8480 | 0.8333 | 0.8344 | 0.8333 |

Supplementary Table S3. Detailed classification performance metrics for brown eggs under Enhance mode (365 nm excitation), including precision, recall, and F1-score

|  | Validation | | | | Test | | | |
| --- | --- | --- | --- | --- | --- | --- | --- | --- |
|  | Precision | Recall | F1 score | Accuracy | Precision | Recall | F1 score | Accuracy |
| ConvNeXt | 1.000 | 1.000 | 1.000 | 1.000 | 0.8770 | 0.8667 | 0.8694 | 0.8667 |
| MobileNet | 1.000 | 1.000 | 1.000 | 1.000 | 0.9099 | 0.9000 | 0.8985 | 0.9000 |
| ResNet-50 | 1.000 | 1.000 | 1.000 | 1.000 | 0.7969 | 0.8000 | 0.7957 | 0.8000 |
| ResNet-101 | 1.000 | 1.000 | 1.000 | 1.000 | 0.8706 | 0.8667 | 0.8665 | 0.8667 |
| DeiT-Base | 1.000 | 1.000 | 1.000 | 1.000 | 0.8118 | 0.8167 | 0.8138 | 0.8167 |
| DeiT-Small | 1.000 | 1.000 | 1.000 | 1.000 | 0.7808 | 0.7833 | 0.7816 | 0.7833 |
| ViT-Base-224 | 1.000 | 1.000 | 1.000 | 1.000 | 0.8735 | 0.8333 | 0.8352 | 0.8333 |
| ViT-Base-384 | 1.000 | 1.000 | 1.000 | 1.000 | 0.8844 | 0.8667 | 0.8633 | 0.8667 |
| ViT-Large | 1.000 | 1.000 | 1.000 | 1.000 | 0.7583 | 0.7500 | 0.7503 | 0.7500 |

Supplementary Table S4. Detailed classification performance metrics for white eggs under Enhance mode (365 nm excitation), including precision, recall, and F1-score.

|  | Validation | | | | Test | | | |
| --- | --- | --- | --- | --- | --- | --- | --- | --- |
|  | Precision | Recall | F1-score | Accuracy | Precision | Recall | F1-score | Accuracy |
| ConvNeXt | 0.8922 | 0.8833 | 0.8839 | 0.8833 | 0.8915 | 0.8667 | 0.8661 | 0.8667 |
| MobileNet | 0.7838 | 0.7833 | 0.7779 | 0.7833 | 0.8015 | 0.8000 | 0.7914 | 0.8000 |
| ResNet-50 | 0.7629 | 0.7667 | 0.7627 | 0.7667 | 0.7406 | 0.7500 | 0.7300 | 0.7500 |
| ResNet-101 | 0.7683 | 0.7500 | 0.7524 | 0.7500 | 0.8457 | 0.8167 | 0.8144 | 0.8167 |
| DeiT-Base | 0.9365 | 0.9333 | 0.9332 | 0.9333 | 0.9176 | 0.9167 | 0.9166 | 0.9167 |
| DeiT-Small | 0.9226 | 0.9167 | 0.9163 | 0.9167 | 0.8511 | 0.8333 | 0.8331 | 0.8333 |
| ViT-Base-224 | 0.9521 | 0.9500 | 0.9493 | 0.9500 | 0.8903 | 0.8833 | 0.8825 | 0.8833 |
| ViT-Base-384 | 0.9185 | 0.9167 | 0.9139 | 0.9167 | 0.9177 | 0.9167 | 0.9166 | 0.9167 |
| ViT-Large | 0.9706 | 0.9667 | 0.9665 | 0.9667 | 0.8939 | 0.8833 | 0.8839 | 0.8833 |

Supplementary Table S5. Comparison of computational complexity for all evaluated models, detailing input resolution, number of parameters (M), and floating-point operations (FLOPs, G).

| Model Architecture | Input Resolution | Parameters (M) | FLOPs (G) |
| --- | --- | --- | --- |
| MobileNet V2 | 224×224 | 3.5 | 0.3 |
| ResNet-50 | 224×224 | 25.6 | 4.1 |
| ResNet-101 | 224×224 | 44.5 | 7.8 |
| ConvNeXt-Base | 224×224 | 88.6 | 15.4 |
| DeiT-Base | 224×224 | 86.6 | 17.6 |
| Deit-Small | 224×224 | 22.1 | 4.6 |
| ViT-Base-224 | 224×224 | 86.6 | 17.6 |
| ViT-Base-384 | 384×384 | 86.6 | 55.4 |
| ViT-Base-Large | 224×224 | 304.3 | 61.6 |
